# Promoting progress in child survival across four African countries: the role of strong health governance and leadership in maternal, neonatal and child health

**DOI:** 10.1093/heapol/czy105

**Published:** 2019-01-29

**Authors:** Connie A Haley, Marie A Brault, Kasonde Mwinga, Teshome Desta, Kenneth Ngure, Stephen B Kennedy, Margaret Maimbolwa, Precious Moyo, Sten H Vermund, Aaron M Kipp, WHO AFRO Child Survival Study Team, Stewart Kabaka, Kibet Sergon, Adolphus T Clarke, Musu C Duworko, Penny Kalesha-Masumbu, Mary Katepa-Bwalya, Bernard Madzima, Trevor Kanyowa, Phanuel Habimana

**Affiliations:** 1 Vanderbilt Institute for Global Health, Vanderbilt University, 2525 West End Avenue, Nashville, TN, USA; 2 Department of Medicine, Vanderbilt University Medical Center, 1161 21st Avenue South, Nashville, TN, USA; 3 Department of Anthropology, University of Connecticut, 354 Mansfield Road, Storrs, CT, USA; 4 World Health Organization, Regional Office for Africa, Cite du Djoue, Brazzaville, Congo; 5 World Health Organization, Inter-country Support Team for East and Southern Africa, Harare, Zimbabwe; 6 School of Public Health, Jomo Kenyatta University of Agriculture and Technology, Nairobi, Kenya; 7 University of Liberia-Pacific Institute for Research & Evaluation (UL-PIRE) Africa Center, University of Liberia, Monrovia, Liberia; 8 School of Medicine, University of Zambia, Lusaka, Zambia; 9 Collaborative Research Program, University of Zimbabwe/University of California, San Francisco, Harare, Zimbabwe; 10 Department of Pediatrics, Vanderbilt University Medical Center, 2200 Children's Way, Nashville, TN, USA; 15 Kenya Ministry of Health, Nairobi, Kenya; 16 WHO/Kenya Country Office, Nairobi, Kenya; 17 Liberia Ministry of Health, Monrovia, Liberia; 18 WHO/Country Office, Monrovia, Liberia; 19 Zambia Ministry of Health, Lusaka, Zambia; 20 WHO/Zambia Country Office, Lusaka, Zambia; 21 Zimbabwe Ministry of Health, Harare, Zimbabwe; 22 WHO/Zimbabwe Country Office, Harare, Zimbabwe; 23 WHO/Regional Office for Africa, Brazzaville, Congo

**Keywords:** Child health, governance, Millennium Development Goals, accountability, health services, qualitative research

## Abstract

Despite numerous international and national efforts, only 12 countries in the World Health Organization’s African Region met the Millennium Development Goal #4 (MDG#4) to reduce under-five mortality by two-thirds by 2015. Given the variability across sub-Saharan Africa, a four-country study was undertaken to examine barriers and facilitators of child survival prior to 2015. Liberia and Zambia were chosen to represent countries making substantial progress towards MDG#4, while Kenya and Zimbabwe represented countries making less progress. Our individual case studies suggested that strong health governance and leadership (HGL) was a significant driver of the greater success in Liberia and Zambia compared with Kenya and Zimbabwe. To elucidate specific components of national HGL that may have substantially influenced the pace of reductions in child mortality, we conducted a cross-country analysis of national policies and strategies pertaining to maternal, neonatal and child health (MNCH) and qualitative interviews with individuals working in MNCH in each of the four study countries. The three aspects of HGL identified in this study which most consistently contributed to the different progress towards MDG#4 among the four study countries were (1) establishing child survival as a top national priority backed by a comprehensive policy and strategy framework and sufficient human, financial and material resources; (2) bringing together donors, strategic partners, health and non-health stakeholders and beneficiaries to collaborate in strategic planning, decision-making, resource-allocation and coordination of services; and (3) maintaining accountability through a ‘monitor-review-act’ approach to improve MNCH. Although child mortality in sub-Saharan Africa remains high, this comparative study suggests key health leadership and governance factors that can facilitate reduction of child mortality and may prove useful in tackling current Sustainable Development Goals.

**Key Messages**

- Stable and consistent health governance and leadership was a key factor contributing to the variable progress towards the Millennium Development Goal Four (MDG#4) target of reducing under-five mortality by two-thirds by 2015.
- Three main aspects of successful health governance and leadership effecting improved child survival identified in this study were (1) establishing child survival as a top national priority backed by a comprehensive policy and strategy framework and sufficient human, financial and material resources; (2) bringing together donors, strategic partners, health and non-health stakeholders and beneficiaries to collaborate in strategic planning, decision-making, resource-allocation and coordination of services; and (3) maintaining accountability through a ‘monitor-review-act’ approach to improve MNCH.
- Countries that made inadequate progress towards MDG#4, struggled to fully support MNCH care, implement policies and strategies, maintain a functional health system, coordinate stakeholders to integrate programmes and services, or ensure effective monitoring and use of health data to identify and overcome gaps in health services.

## Introduction

Substantial progress in child survival led to an estimated decline in under-five mortality (U5M) worldwide from 12.7 million in 1990 to 5.9 million in 2015 (UNICEF *et al*., 2015). However, progress was limited in many regions, such that Millennium Development Goal #4 (MDG#4) to reduce global U5M by two-thirds between 1990 and 2015 was not met (United Nations, 2000). Although the rate of U5M in sub-Saharan Africa (SSA) remains the highest in the world, estimated at 83 deaths per 1000 live births in 2015 (UNICEF *et al*., 2015), 12 SSA countries met their MDG#4 target: Eritrea, Ethiopia, Liberia, Madagascar, Malawi, Mozambique, Niger, Rwanda, Senegal, Tanzania, Uganda and Zambia (UNICEF *et al*., 2015; You *et al*., 2015). These successes demonstrate that substantial reduction in childhood deaths is possible in low- and middle-income countries (LMICs).

Most childhood morbidity and mortality can be prevented or cured with known, affordable technologies and treatments (Friberg *et al*., 2010; Berman *et al*., 2016; Moucheraud *et al*., 2016). Yet, inadequate health systems in many LMICs hinder progress such that essential drugs and interventions are not distributed reliably, in sufficient quantity, equitably or at reasonable cost. Published case studies highlight how some SSA countries accelerated progress to reduce U5M, providing valuable insights regarding implementation and scale-up of child survival strategies (Bellagio Group, 2003; Amouzou *et al*., 2012; Mbonye *et al*., 2012; Zimba *et al*., 2012; Kuruvilla *et al*., 2014; Afnan-Holmes *et al*., 2015; Requejo *et al*., 2015; United Nations, 2015; Kanyuka *et al*., 2016; Moucheraud *et al*., 2016; Ruducha *et al*., 2017), but few comprehensively evaluate countries making insufficient progress towards MDG#4.

**Figure 1:**
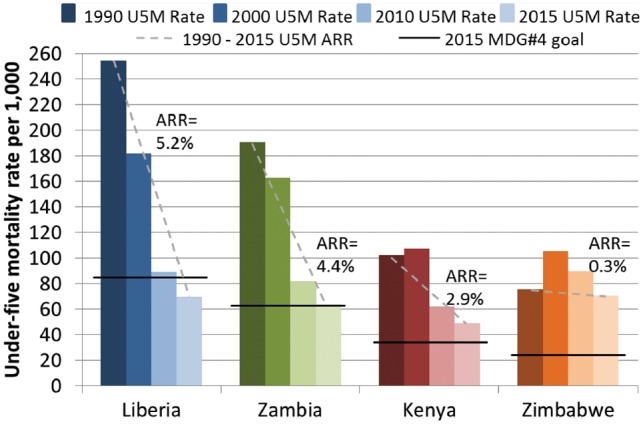
Trends in under-five mortality and progress towards Millennium Development Goal #4 for Liberia, Zambia, Kenya and Zimbabwe, 1990–2015

We previously conducted country-specific case studies of four SSA nations with different annual rates of reduction (ARR) in U5M to identify specific barriers and facilitators that influenced their progress towards MDG#4 (Figure 1) (Kipp *et al*., 2016; Brault *et al*., 2017, 2018; Haley *et al*., 2017). Liberia and Zambia were on track for MDG#4 when the study began (and have now met MDG#4) while Kenya and Zimbabwe were not on track (and did not meet MDG#4) (Figure 1). Country trends for infant mortality mirrored those of U5M. Neonatal mortality declined by ∼50% for Liberia and Zambia, yet remained stagnant for Kenya and Zimbabwe. Each country has unique historical, social and political experiences, while sharing characteristics such as high poverty levels, developing economies and large rural populations (see Boxes 1–4). Notably, we identified health governance and leadership (HGL) as a factor influencing progress in reducing U5M in these four countries.

**Box 1 Historical and contextual factors impacting Liberia during the study period**

- Located on the western coast of Africa with a small population of about 3.5 million, half of whom reside in urban areas
- Fourteen years of civil war ending in 2003 destroyed most of the national infrastructure, internally displaced many people and cost at least 200 000 lives
- First democratic election held in 2005 began a new phase of national reforms and rebuilding
- Prudent macroeconomic management, social stability and substantial foreign investments have facilitated efforts to overcome the civil crisis and re-establish its economy
- Economic growth affected by infrastructure constraints, unemployment, a narrow base of the economy and the country’s dependency on food and fuel imports
- Compounding challenges include flooding and drought in some areas, outbreaks of communicable diseases, influx of more than 150 000 refugees from neighbouring Cote D’Ivoire and increasing dependence on international aid

**Box 2 Historical and contextual factors impacting Zambia during the study period**

- Gained independence in 1964, has since enjoyed decades of political stability and freedom from conflict enabling a consistent focus on development and reforms
- Experienced consistent economic growth and strong macroeconomic indicators over several decades
- Growing population, high level of urbanization and increasing life expectancy
- Young and increasing population also intensifies the burden of health needs on the economy
- Economic growth has not translated into significant poverty reduction at household level; more than half of the population lives below the poverty line, most considered to be in extreme poverty
- Unemployment has been high and income inequality is significant

**Box 3 Historical and contextual factors impacting Kenya during the study period**

- Largest and most diversified economy in East Africa
- Strategically located to serve as an important transport hub for much of Eastern Africa
- Large and growing population consisting of most major ethno-racial and linguistic groups found in Africa
- High absolute poverty; more than two-thirds of urban population living in slums
- Violence following the 2007 presidential elections worsened mistrust between different political and ethnic groups
- Decades of globalization, political instability, regional and national macroeconomic challenges and climate change have contributed to high inequities

**Box 4 Historical and contextual factors impacting Zimbabwe during the study period**

- Overcame a decade of civil war to gain independence in 1980, and successfully established one of the strongest economies and health systems in southern Africa
- Long period of relative stability and progress following independence until it experienced a drastic economic decline and hyperinflation beginning in the late-1990s
- Prior to the economic crisis, Zimbabwe had a highly performing health delivery system supporting a long track record of delivering comprehensive health services across the country.
- Nearly a quarter of the population left the country, including a large proportion of the workforce
- High poverty rates, unemployment and food insecurity persisted during the study period
- Despite challenges, education and literacy rates remained high among both men and women

HGL has been defined in different ways in the literature (Barbazza and Tello, 2014; Mishra *et al*., 2015; Moucheraud *et al*., 2016). We used the World Health Organization’s (WHO) definition which provides a practical country-level framework for HGL: ‘ensuring strategic policy frameworks exist and are combined with effective oversight, coalition-building, regulation, attention to system-design and accountability’ (WHO, 2007). Widely considered the most critical of WHO’s health system building blocks, HGL links all health system components together, providing strategic direction for ensuring availability of high quality health services, managing the health workforce, providing medicines, financing health services and generating information needed for effective decision-making (WHO, 2007; Cavagnero *et al*., 2008). In this study, we re-analysed data from all four country case-studies, including a review of national policies and strategies pertaining to the larger scope of maternal, neonatal and child health (MNCH) under which U5M falls, and qualitative interviews with individuals working in MNCH to elucidate specific components of HGL that influenced achieving (Liberia and Zambia) or not achieving (Kenya and Zimbabwe) MDG#4.

## Methods

We reviewed national policies and strategies issued between 2000 and 2013 and conducted key informant (KI) interviews in 2013 to explore eight content areas influencing child survival (WHO, 2006, 2007, 2010, 2012; Ban, 2010; WHO and PMNCH, 2011): (1) health care system (including HGL, structure, human resources for health, access & utilization, monitoring & evaluation and accountability), (2) national health strategies and policies, (3) MNCH interventions, (4) clinical standards and guidelines, (5) commodities and essential medicines, (6) health financing, (7) partnerships and (8) contextual factors (e.g. conflict, political environment, hygiene and sanitation, nutrition and food security, education and human rights).

Four SSA countries (Liberia, Zambia, Kenya and Zimbabwe) were chosen based on their U5M ARR between 1990 and 2011 (data available when the study was designed, Figure 1) and their national governments’ willingness to participate. Detailed study methods for each country case study have been published (Kipp *et al*., 2016; Brault *et al*., 2017, 2018; Haley *et al*., 2017).

### Review of MNCH policies and strategies

A national document review was conducted for each country to evaluate the MNCH policy framework affecting progress towards MDG#4. Policies and strategies pertaining to overall national health, MNCH and other related determinants were obtained from the WHO African Region office, WHO country focal points and Ministry of Health (MOH) for Liberia, Zambia, Kenya and Zimbabwe. Additional MNCH-related documents referenced in initial sources were subsequently obtained and reviewed (see individual case study supplementary tables in Kipp *et al*., 2016; Brault *et al*., 2017, 2018; Haley *et al*., 2017).

An abstraction guide was developed based on the eight study content areas and several cross-cutting questions (Table 1). Each document was reviewed by one author (CAH), who consulted with a second reviewer (MAB) as needed. Information from original documents was recorded verbatim in the abstraction guide to avoid observer bias.

**Table 1:** Key questions and deductive themes explored during the review of national health policies and strategies and key informant interviews that cut across child survival content areas

Specific questions for review of national policies and strategies	Specific themes explored across content areas with key informants
What policies and strategies related to MNCH were in place between 2000 and 2013 (including changes during this period)? What challenges were stated as hindering progress towards MDG#4? What facilitators were stated as enabling progress towards MDG#4? What changes or improvements to MNCH policies and strategies were proposed or newly implemented towards the end of the study period but were not yet measurable?	Issues related to programme evaluation, access and utilization, coverage, impact and sustainability, as appropriate Knowledge and experiences related to MNCH across the health care continuum (prenatal care through age 5 years) Knowledge and experiences related to MNCH across the health system continuum (community to tertiary hospitals)

### Qualitative methods

#### Study location and participants

Utilizing country Demographic and Health Surveys (DHS) closest to 1990 and 2011, one or two provinces were selected from each country that had U5M ARRs comparable with the national ARR and were logistically accessible. Specific rural and urban sites were selected to evaluate differences in MNCH that can exist between urban and rural areas (Table 2).

**Table 2:** Selected study sites within Kenya, Liberia, Zambia and Zimbabwe

Country	Capital	Urban	Rural
Kenya^a^	Nairobi (Nairobi Province)		Embu (Eastern Province)
Liberia	Monrovia (Montserrado County)		Gbarnga (Bong county)
Zambia	Lusaka	Livingstone (Southern Province)	Kazungula (Southern Province)
Zimbabwe	Harare	Chinhoyi (Mashonaland West Province)	Banket (Mashonaland West Province)

a Nairobi Province is now Nairobi County; Eastern Province now consists of eight counties (established in 2013), including Embu County as the rural study site.

#### Study participants

Semi-structured interviews were conducted with KIs involved in MNCH from the MOH, donor organizations, community-based organizations (CBO) and health care providers (HCP) (Tables 3 and 4). CBO participants and HCPs were selected from both urban and rural sites. National level KIs (see below) were recruited from the capital and each local site. In-country research teams collaborated with the MOH and WHO to identify potential KIs representing a range of ages, work experiences and positions/roles balanced between urban and rural sites.

**Table 3:** Additional inclusion criteria for each key informant group

Key informant type	Description
All participants	Age 18 years or older Have adequate knowledge or experiences related to childhood survival specified for each participant group below Speak English or the most common local language, Able to provide written or verbal informed consent.
Ministry of Health	National or provincial-level officials working in government-level health care system administration, policy-making, programme development or leadership. All officials working in areas related to MNCH were eligible.
Donor partners	Individuals working as directors, managers or other leaders of entities providing financial or other aid for MNCH services, or serving as the implementing partner. International or national organizations focusing entirely on MNCH or with MNCH as one component of their mission. Organizations had to be officially registered in the country.
Members of community-based organizations	Directors, leaders, managers working for a CBO involved in or providing referrals to MNCH services within the study site. Organizations had to be officially registered in the country.
Health care providers	Professionally trained physicians, nurses, clinical officers or other health-related staff such as environmental health technicians, pharmacists or community health workers. Working in a health facility providing MNCH care.

**Table 4:** Numbers of key informants interviewed for each country

	Ministry of Health	Donor organization	Community-based organization	Health care worker	Total
Kenya	9	8	13	13	43
Liberia	11	8	14	14	47
Zambia	6	6	10	9	31
Zimbabwe	6	6	6	12	30
Total	32	28	43	48	151

#### Data collection and analysis

Guides for KI interviews were developed and piloted, mirroring the eight content areas and cross-cutting questions explored in the national document review (Table 1). Interviews were audio recorded, transcribed and translated into English (as needed) by trained research assistants. Transcripts were coded using deductive themes based on study content areas plus additional themes identified upon transcript review. Analyses were conducted using the qualitative software Atlas.ti (Murh, 2004), grouping the on-track countries (Liberia and Zambia) and not on-track countries (Kenya and Zimbabwe) for comparison. Analyses focused on codes related to HGL based on the WHO definition (WHO, 2007).

The Institutional Review Boards at the authors’ institutes and both the national and local ethics and research committees for each country approved the qualitative component of the study as follows (see Supplementary file S1 for copies of approval letters): Vanderbilt University Medical Center (Coordinating Center), Kenyatta National Hospital Ethics & Research Committee (Kenya), University of Liberia Office of the Institutional Review Board (Liberia), ERES Converge Institutional Review Board (Zambia), Joint Parirenyatwa Hospital and University of Zimbabwe College of Health Sciences Research Ethics Committee and the Medical Research Council of Zimbabwe.

## Results

### Liberia

#### Prioritization and support of child survival

National documents and KIs described Liberia’s focused efforts to rebuild the healthcare system and establish essential services following a prolonged civil crisis. A strong policy framework was devised and implemented, including a triple planning approach using immediate, short- and long-term plans concurrently focusing on health, social welfare and development [Liberia Ministry of Health and Social Welfare (MoHSW), 2008; MoHSW, 2011d; Liberia Ministry of Planning and Economic Affairs (MPEA), 2012]. Liberia’s first post-conflict national health policy and strategic plan (MoHSW, 2007) prioritized MNCH through primary health care, community empowerment and cross-sectoral partnerships. Within 5 years, Liberia updated its national policies, integrating health and social determinants to increase equitable access to comprehensive packages of MNCH services delivered closer to communities (MoHSW, 2011b). Nearly all KIs felt these policies spearheaded by Liberia’s president enabled rapid recovery of the health system and increased utilization of MNCH services.

*… the President had launched the revised road map for accelerating the reduction of maternal mortality, maternal and newborn mortality and morbidity in Liberia … initiatives that we believe … [have] shown government own commitment …* (49-year-old male donor partner)*.*

With significant donor support for overall development, Liberia increased total government expenditure on health (TGEH) to exceed the Abuja Declaration target of at least 15% of a country’s annual budget [African Union (AU), 2006]. National documents and KIs reported that resources supporting MNCH were generally allocated appropriately and directed towards high priority areas, but that additional government funding was needed to fully implement MNCH interventions.

#### Collaboration, coordination and inclusion

National documents and KIs asserted that Liberia’s government developed collaborative multi-sectoral partnerships at all levels of the health system, aligning local MNCH activities with national priorities (MoHSW, 2011d). A 2009 decentralization policy shifted health services funding and allocation decisions to sub-national leaders more knowledgeable about local needs. In addition, a 2011 community health services policy established services closer to the populations in need (MoHSW, 2011c). Moreover, KIs felt the government effectively coordinated international donors, national and local programme leaders and community health providers and beneficiaries, to integrate delivery of MNCH services at each point of care. Although Liberia maintained programme-specific policies and strategies (e.g. for HIV/AIDS, malaria, immunization and food security), the Ministries of Child Health and Social Welfare were merged to enable a holistic approach to MNCH, which was viewed favourably by KIs.

*… it [effective external partnerships] was develop[ed] through coordination meetings …. When they came, some of them started doing their own thing …. But when the Ministry of Health said we have to meet and coordinate and know exactly what each partner is doing … people started coordinating and started working together and looking at best practices and start planning to have one focus …* (50-year-old male urban CBO partner)*.*

*[W]henever there is a new policy in place, the ministry will inform the county health team, they will do trainers of trainers, from county level, district level and then they will train facility level staff to implement these policies and then down to the community level* (49-year-old female donor partner).

#### Accountability

Liberia set specific health targets, timeframes, roles and responsibilities within its child health policy framework aimed specifically at reaching MDG#4. An effective national and district-level health management information system (HMIS) enabled reporting of surveillance data, vital statistics and health services data from local facilities and providers up to county and national levels. National documents (MoHSW, 2011a) and KIs described timely collection and review of data as facilitating ongoing monitoring, evaluation and data-driven decision-making. County health and social welfare boards and community health committees further encouraged stakeholder and community involvement in HGL and ensured accountability for MNCH resource allocation.

*… the Ministry of Health and Social Welfare bases its plans on evidence; every activity, every move to improve [MNCH] is based on data, based on situations analysis that was conducted and high impact interventions identified to affect situations …. They are constantly monitored, evaluated and discussed and reviewed …* (45-year-old male MOH representative).

*One other thing that promoted the effective partnership was accountability. Because we started understanding that if somebody gives you money, to give back you need to give account …. Nobody wants to give somebody something who doesn’t know his left hand from his right hand …* (48-year-old male urban CBO partner).

### The Republic of Zambia

#### Prioritization and support of child survival

Zambia’s achievements in MNCH and health sector reforms steadily evolved over decades of political stability with a commitment to reducing U5M by focusing on immediate, medium- and long-term goals. Health system restructuring was intentionally aligned with development and poverty reduction efforts through five consecutive National Health Strategic Plans, six corresponding National Development Plans and a long-term National Development Strategy [Zambia Ministry of Health (MOH), 2006, 2012]. Zambia prioritized reduction of U5M through a comprehensive health policy framework that reflected international recommendations and resolutions related to MNCH [Zambia MOH, 2012; Zambia Ministry of Community Development and Mother and Child Health (MCDMCH) and MOH, 2013]. Expanded access to MNCH care was facilitated through a policy to remove user fees, adoption of a ‘Primary Health Care Approach’ (WHO, 2008) and delivery of integrated packages of basic health services from pregnancy thorough adolescence and across health system levels (Zambia MOH, 2012). In 2011, MNCH services were moved into an expanded Ministry of Community Development, Mother and Child Health to holistically address poverty, health and other social welfare issues. In addition, Zambia’s Constitution was amended to guarantee children’s right to health, and the government strengthened its policy framework to improve newborn health and provide a roadmap for achieving MDG#4 (MCDMCH, 2013b; MCDMCH and MOH, 2013).

KIs described a well-structured national system for identifying and funding local MNCH priorities and needs and expanding community-level services. Though TGEH was increased to meet the Abuja Declaration (Countdown to 2015, 2012; USAID and UNAIDS, 2013), some KIs felt that additional government funding was needed to avoid reliance on donors.

*… more and more efforts are being made towards maternal and child health in terms of trying to increase funding and trying to make those facilities available and accessible and … now [the] creation of a new ministry which entirely looks at the mother and child health so that … it is prioritized …* (51-year-old male MOH representative).

*… even if we have very few resources, we prioritize it, that finances at least should go to maternal and child health* (43-year-old male urban healthcare provider).

#### Collaboration, coordination and inclusion

Zambia’s well-structured health system and MNCH policy framework promoted strong partnerships with external donors willing to align their support with domestic priorities. According to national documents and KIs, the government’s collaborative approach and decentralized HGL facilitated partnerships among health sector departments, between health and non-health ministries, and with a diversity of stakeholders at national and local levels (Zambia MOH and WHO, 2011; Zambia MOH, 2012). Local stakeholders were engaged in the coordination and integration of MNCH services, through an Interagency Coordinating Committee and technical working groups used to identify gaps, remove bottlenecks, mobilize resources and improve efficiency.

*… we have a sectorial advice group meeting and these are platforms that we use to try and persuade partners to buy into the health sector strategic plan … instead of them dreaming up something that they want to do, we actually present the activities that we have included in the strategic plan … [with] some partner input in them* (43-year-old male MOH representative).

*They’ve known … that they need to have a community led strategy of people mobilizing fellow community members to go and have vaccinations so they … have what they call reaching every child … where they try to promote community efforts in supporting the program …* (41-year-old female donor partner).

#### Accountability

Per national documents and KIs, Zambia fostered accountability throughout the health system by conducting ongoing and effective monitoring and evaluation efforts while encouraging feedback from stakeholders and beneficiaries. This process was facilitated by a highly functioning HMIS (Zambia MOH, 2013) and effective oversight of national electronic reporting for vital statistics, disease surveillance and response, human resources, pharmaceutical supply and distribution and finance and administration. An innovative electronic health records system was established to feed directly from the point of care into the HMIS, allowing detailed and timely reporting of MNCH service utilization, health expenditure and clinical outcomes (MCDMCH, 2013a). The data informed strategic planning, resource allocation and quality improvement, which along with a Zambian-led Countdown to 2015 initiative, accelerated achievement of MDG#4 (Zambia MOH, 2008).

*First and foremost, it’s identifying and having the right mix of priorities so in the development of the national health strategic plan … we use available data, mortality data, service data to look at where the need is greatest …* (43-year-old male MOH representative)*.*

*… Zambia is among very few countries who have done impact studies for a number of good years. To see how we are progressing, how those interventions we are employing whether they are working or not …* (51-year-old male MOH representative).

### Kenya

#### Prioritization and support of child survival

During most of the study period, inadequate investment in the national health system led to stagnating public health sector performance, worsening health inequities and reversals of previous gains in child health outcomes [MOPHS, 2008; Kenya Ministry of Medical Services (MOMS) and Ministry of Public Health and Sanitation (MOPHS), 2012]. The government of Kenya also underwent several transitions, including a period of marked instability following the 2007 elections. Corresponding changes occurred in national HGL, with the MOH dividing into separate Ministry of Public Health and Sanitation (MOPHS, responsible for primary care at the community, dispensary and health centres levels) and Ministry of Medical Services (MOMS, responsible for the highest system levels) in 2008 before being re-unified in 2013.

*We thank God that now the MOMS and the MOPHS have come together, that is also what was causing a lot of division … [MOPHS] had a lot of resources than the MOMS, but now it is integrated …* (57-year-old female urban healthcare provider).

Kenya’s comprehensive national MNCH policy framework was described by both national documents and KIs as largely ineffective during most of the study period. One document described ‘years of erratic application of policy’ and ‘inadequate financial and human resources, inefficient support systems, and poorly coordinated responses to public health problems’ leading to poor health system performance (MOPHS, 2008). Later in the study period Kenya renewed its focus on health system strengthening and the right to health through a long-term national development plan (Government of Kenya, 2007) and a new Constitution (Government of Kenya, 2010), but progress was hindered by unresolved short-term challenges. Devolution of HGL to sub-national levels aimed to improve service delivery, accountability, citizen participation and equitable resource distribution, but this was not achieved during the study period. An updated National Health Sector Strategic Plan was issued to expand equitable access to care and strengthen community-level interventions through the Kenya Essential Package for Health (KEPH) and a Community Health Strategy (CHS) [Kenya Ministry of Health (MOH), 2007; MOMS *et al*., 2009; MOMS and MOPHS, 2013]. However, implementation was described as ‘slow’, and limited by inadequate human resources in many areas [National Coordinating Agency for Population and Development (NCAPD) [Kenya] *et al*., 2011]. Comprehensive strategies targeting newborn survival and U5M were also developed (MOPHS, 2008; MOPHS and MOMS, 2010), as were policies supporting adequate housing, nutrition, clean water, social security and education (MOMS and MOPHS, 2013). Unfortunately, as one KI stated, ‘[Kenya has] many strategic plans…the problem has been the strategies are there but the implementation is not there’ (40-year-old female urban healthcare provider). In 2012, Kenya’s National Health Policy was revised, promoting a ‘health in all policies’ approach to concurrently address all determinants of health. This revision’s effect could not be determined by the end of the study period (MOMS and MOPHS, 2012, 2013).

National documents and KIs reported chronic government underfunding of Kenya’s health system and MNCH specifically, with nearly all KIs describing limited financial, material and human resources, particularly for primary care. Moreover, donor support was largely project-oriented and not necessarily aligned with Kenya’s priorities (NCAPD Kenya *et al*., 2011). Some KIs reported that the most successful MNCH programmes during the study period were those with steady funding from both the government and external partners.

*… And the government signed the Abuja Declaration to be able to fund health with at least 15% of the national budget. We’ve never gone beyond a 1/3rd of that budget that’s why we’re still struggling …* (53-year-old male MOH representative).

*… [Priorities] seem to change unfortunately depending on where the funds have come from … [W]here the funds are from for HIV services, the HIV gets precedence. If you have a donor who says they want to look at TB, they’ll concentrate on TB, when Malaria, it’s that.…* (37-year-old male donor partner).

#### Collaboration, coordination and inclusion

National documents indicated that persistently centralized HGL led to poor coordination between health system levels and inequitable distribution and financing of health services. KIs, however, expressed optimism that the recent devolution might alleviate this problem. An inter-ministerial National Council for Maternal and Child Health was created to harmonize national policy formulation, planning and coordination, resource mobilization, intervention delivery and monitoring and evaluation but was given no regulatory authority (MOMS and MOPHS, 2013). According to KIs, the lack of coordination, oversight or inclusion of beneficiaries in planning contributed to service gaps, duplication and poor quality of care.

*… there’s been very poor connection or cross sharing of skills, of resources to ensure continuum of care at a service delivery level … the HIV program came in and set up … a vertical PMTCT service in a health system where we had an MCH service and we would have easily integrated that within the MCH. There [is] lots of verticalization including of reporting and of monitoring …* (41-year-old male urban healthcare provider).

*… an unfortunate thing is [in] this country people have been operating in silo[s] … so everybody operating independently …. Probably even one thing in improving child survival is making sure that all of you have the same goal, seeing … what can you complement each other to achieve the same goal or even at a lower cost* (40-year-old female urban CBO partner).

#### Accountability

National documents described health sector ‘accountability deficits’ as contributing to inadequate MNCH service delivery, considerable inequities and poor health outcomes (MOPHS, 2008). Moreover, the country’s weak HMIS limited capacity for compiling, analysing and applying data to improve MNCH programmes or inform health policy (MOMS and MOPHS, 2009; NCAPD Kenya *et al*., 2011).

*Once we implement we need to have a way of having continuous monitoring and evaluation to see where we are at, what impact have we had, so that once an intervention is in place, we are able then to keep upgrading it …* (40-year-old female urban healthcare provider).

*… in Kenya, a bulk of patients are seen in the private sector … we have to strengthen the M and E [monitoring and evaluation] system for all the sectors, whether public or private. We must get them somewhere they are analysed so that we can get the true picture [of the burden of disease]* (50-year-old male urban healthcare provider).

Later national health policies and strategies (MOMS and MOPHS, 2012, 2013) began to strengthen Kenya’s capacity to collect and apply local health data to improve availability and quality of MNCH services. Health management teams and local stakeholders (MOMS and MOPHS, 2009) were tasked with regular performance reviews, and mechanisms were implemented to improve public transparency and accountability. KIs did not discuss these reforms, making it difficult to determine their impact.

### Zimbabwe

#### Prioritization and support of child survival

Following independence in 1980 and a decade of civil war, Zimbabwe developed one of the strongest health systems in southern Africa, achieving lower U5M rates and higher coverage of MNCH interventions compared with other SSA countries. However, national documents and KIs described how Zimbabwe’s health system collapsed following the national socioeconomic crisis that began in the 1990s and peaked in 2009–2010 [Zimbabwe Ministry of Health and Child Welfare (MOHCW), 2010a,b]. Provision of MNCH services at that time was undermined by debilitated health infrastructure, a poorly functioning patient referral system, drug shortages and unaffordable out-of-pocket health care costs. Nearly all KIs and national documents stated that Zimbabwe’s critical shortage of health workers affected quality and availability of MNCH services [MOHCW, 2010b; Osika *et al*., 2010; Zimbabwe Ministry of Economic Planning and Investment Promotion (MEPIP) and United Nations Development Program (UNDP), 2012]. Health management was severely weakened by high attrition rates of experienced leaders, supervisors and programme managers. National health and re-development strategies addressing these limitations were not adequately implemented or funded (MOHCW, 2010b; Osika *et al*., 2010).

*… quality of maternal child born services … at all levels was highly compromised, it was very much substandard. It had something to do with shortage of human resources, had to do with WHO’s shortage of supplies and of course it had something to do with poo[r] supportive supervision and monitoring …* (58-year-old male donor partner).

*… [W]here a nurse knows that I should manage … a sick child using their IMSI protocol but because there is a queue there … and there is just one nurse, they just do a shortcut …* (52-year-old female donor partner).

In the late 2000s, the government of Zimbabwe renewed its commitment to ‘kick-start’ the national health care system and re-focus on national development (MOHCW, 2007, 2010a,b). Zimbabwe’s 2009 National Health Strategy reinstituted measures to improve child survival such as the Primary Health Care Approach (WHO, 2008), delivery of MNCH intervention packages for all life stages at all health system levels, and community health services and outreach activities, but the overarching health policy framework remained outdated.

To increase availability and utilization of MNCH services, Zimbabwe established a user fees exemption policy for the poor and vulnerable (including children) and a 5-year (2011–2015) multi-donor pooled Health Transition Trust Fund to enable health system improvements and increase access to care for mothers and young children. However, TGEH remained far below the Abuja recommendation, and many KIs felt that donor support was unsustainable.

*There is a challenge [that the money] allocated in health ministries [is] very low …. The strongest that has been funding the MNCH is the …. Health Transition Fund, but it has also a limit of … five years and then it goes* (52-year-old female donor partner)*.*

Even at the end of the study period, KIs at various system levels felt that national strategies and policies related to MNCH were generally ‘good on paper’ but were not implemented, coordinated or enforced.

*l think we have the … RH [reproductive health] road map, the RH policy, the child survival strategy … l don’t think there is a serious problem with the policy and strategy, the major problem is translating these strategies and policies into action* (58-year-old male donor partner).

#### Collaboration, coordination and inclusion

Although once decentralized, Zimbabwe’s HGL shifted towards national control over decision-making and resource allocation. This resulted in poor communication with local levels and ‘non-involvement of communities in health planning and management’ (MOHCW, 2010b; Osika *et al*., 2010). Health was considered a sectoral issue instead of a national priority integrated across policies (MOHCW, 2010b). Child Health and Maternal/Reproductive Health were separate departments within the Ministry of Health and Child Welfare (MOHCW), each coordinated by different officers with different reporting hierarchies (MOHCW, 2010a). Poorly synchronized health strategies, limited collaboration and ill-defined roles and responsibilities among stakeholders led to fragmented MNCH programmes and services (MOHCW, 2010b). Development of the National MNCH Steering Committee, National Child Survival Technical Working Group and National Child Welfare Council were intended to promote a participatory leadership structure, but these entities were described as ‘weak’, with limited stakeholder participation (MOHCW, 2010a,b). KIs also expressed concern that nearly every aspect of the MNCH system required the support of external partners, whose priorities were inconsistently aligned with the MOHCW. Vertical approaches intensified uneven distribution of aid and magnified inequities among programmes, populations and geographic areas. Heavy reliance on programme- or condition-specific donor aid also hindered the ‘supermarket approach’ intended to provide multiple MNCH services at one visit (MOHCW, 2010b; MEPIP and UNDP, 2012).

*I think the Ministry needs to continue discussing with lower levels of the health care system so that they understand what is it that is happening at [the] clinic level, and that the national level goes and procure things which cannot be used at clinic level that is a waste of resources …* (60-year-old female donor partner).

*… if you go to a district you find there are a number of donors but if you go to the other, there is not even a single donor. I think the coordination, if possible at national level, should be improved so that there is an equitable distribution of services …* (46-year-old female MOH representative)*.*

#### Accountability

National documents frankly described Zimbabwe’s insufficient progress towards MDG#4 and other health goals, acknowledging limited public availability of health financing and service information and a failure of health committees to involve stakeholders (MOHCW, 2010b). Zimbabwe’s National HIMS was described as ineffective with inadequate oversight resulting in poorly harmonized monitoring and evaluation. More recent national documents noted Zimbabwe’s commitment to accountability, and KIs recognized efforts to improve health data to more effectively track indicators associated with MNCH.

*… you find that there are so many strategic documents, there is HIV/AIDS, MNCH, RH, so they are there but they are not integrated so you find each one will come up with their own M and E systems and they are donor driven programmes …* (60-year-old female donor partner).

*… we are also trying to support the monitoring evaluation system including the … national health management of information system …. Now the provinces have restarted conducting their own planning review meetings every six months …[and] now the quality has started improving …* (58-year-old male donor partner).

### Cross-country summary

Table 5 summarizes the similarities and differences in the HGL themes described above for each country. Overwhelmingly, Liberia and Zambia successfully engaged with or implemented these elements during the study period. In contrast, Kenya and Zimbabwe struggled to do so, despite sometimes having the appropriate frameworks or approaches.

**Table 5:** Comparison of health governance and leadership elements between progressing and non-progressing countries

	Progressing		Non-progressing	
	Liberia	Zambia	Kenya	Zimbabwe
Prioritization and support of child survival				
Political support	+	+	+/−	+/−
Current policy framework	+	+	+	−
Policies and strategies implemented	+	+	−	−
Concurrent national policy focus on health, social welfare, development	+	+	−	−
Triple planning approach	+	+	+/−	−
Abuja Declaration target met during study	+	+	−	−
Non-financial health system resources (human, material, facility, etc.)	+	+	−	−
Collaboration, coordination and inclusion				
Donors aligned with national priorities	+	+	−	−
Collaborative strategic planning with partners/stakeholders	+	+	−	−
Coordination/collaboration between health and other sectors	+	+	−	−
Coordination and sharing resources among different health programmes	+	+	−	−
Coordination of MNCH services across health system levels	+	+	−	−
Integrate packages of health services at point of care	+	+	−	−
Decentralization of decision-making and resource allocation	+	+	−	−
Beneficiaries included in strategic planning (community input)	+	+	−	−
Accountability				
Clear roles, responsibilities and expectations	+	+	+/−	−
Updated, effective HMIS	+	+	−	−
Consistent data collection and reporting at all health system levels	+	+	−	−
Ongoing monitoring and evaluation of health programmes and interventions	+	+	−	−
Specifically monitoring of progress towards MDG#4	+	+	+/−^a^	+/−
Data-driven planning and decision-making responsive to population needs	+	+	−	−
Local involvement (community planning boards and committees)	+	+	−	−

+ Indicates clear activity, policy, participation and/or implementation of an element in the defined area during the study period; − indicates a lack of engagement of this element or merely planning, but not implementing policy/action during the study period; +/− Indicates ambiguous activity, policy, participation and/or implementation of an element in the defined area.

a We found information indicating that a Kenya Country Countdown was conducted in 2013 (end of the study period), though this was not reported to our study team by Kenya’s MOH.

## Discussion

Among the four study countries, Liberia and Zambia reduced U5M by two-thirds between 1990 and 2015, but both had almost double the U5M rates of Kenya and Zimbabwe in 1990. While slower progress in Kenya and Zimbabwe could have been influenced by the complexities of reducing preventable child deaths when starting at a lower baseline, this cross-study analysis identified HGL as a notable factor contributing to the differences in progress among study countries. Other published case studies from LMICs have also identified strong country HGL as a success factor for reducing U5M (Amouzou *et al*., 2012; Kuruvilla *et al*., 2014; Ahmed *et al*., 2016; Huicho *et al*., 2016; Kanyuka *et al*., 2016; Moucheraud *et al*., 2016; Ruducha *et al*., 2017). Effective HGL enables a solid health system foundation of national management capacity, comprehensive legislation, well-equipped workforce, functioning infrastructure, sufficient funding and robust data for decision-making, transparency and accountability. Our study expanded on these prior findings by identifying three overarching components of HGL that influenced progress in reducing U5M: (1) establishing child survival as a top national priority backed by a comprehensive policy and strategy framework and sufficient human, financial and material resources; (2) bringing together donors, strategic partners, health and non-health stakeholders and beneficiaries for strategic planning, decision-making, resource-allocation and coordination of services; and (3) maintaining accountability through a ‘monitor-review-act’ approach to improve MNCH.

Liberia and Zambia clearly established child survival as a top priority supported by updated policy frameworks aligned with international recommendations and financed at the globally recommended level (African Union, 2006). Both countries integrated the health sector’s strategic direction with social welfare and development rather than having disconnected plans competing for attention and resources (Cavagnero *et al*., 2008; United Nations, 2010). Moreover, both Liberia and Zambia highlight the benefit of a ‘triple-planning approach’ in MNCH policy development, synchronously addressing urgent needs, adapting mid-term strategies to accelerate progress while also implementing sustainable long-term approaches, as shown in other countries achieving MDG#4 (Kuruvilla *et al*., 2014). High coverage of MNCH services has consistently been linked to cross-sector efforts addressing poverty, nutrition, education, gender equity, disease and sanitation (WHO and UNICEF, 2013; Mishra *et al*., 2015; Rasanathan *et al*., 2015), and approximately half of the reduction in maternal and child mortality in LMICs since 1990 is attributable to non-health sector investments (Kuruvilla *et al*., 2014; Bishai *et al*., 2016). While U5M can be reduced by leveraging limited resources across health programmes and other sectors (Ban, 2010; Jamison *et al*., 2013; Stenberg *et al*., 2014; Lie *et al*., 2015; Mishra *et al*., 2015), strong health systems require sustained investment (Every Woman Every Child, 2015). In contrast, persistently low health financing in Kenya and Zimbabwe hindered implementation of MNCH-related policies and strategies (Mishra *et al*., 2015). HGL in these countries remained focused on more immediate obligations and challenges rather than longer-term health system reforms.

Collaborative partnerships offer LMICs a vehicle for aligning interests and obtaining additional resources to implement MNCH initiatives. However, strong HGL is required to effectively coordinate partners across the health system and to align donor assistance with national priorities (Organisation for Economic Co-operation Development, 2005; Atun *et al*., 2011; Mishra *et al*., 2015). HGL in both Liberia and Zambia collaborated with partners for strategic planning and persuaded them to support government-established MNCH initiatives. This balancing of donor investment in specific health interventions with more general health system strengthening can increase availability of health services (Kinney *et al*., 2010; Bryce *et al*., 2013; WHO and PMNCH; 2013; Stenberg *et al*., 2014; Mishra *et al*., 2015). In addition, a ‘health in all policies’ approach with integration of health and non-health programmes enabled HGL in Liberia and Zambia to synergize the efficient and effective provision of MNCH services (Kerber *et al*., 2007; Friberg *et al*., 2010; Were *et al*., 2015). Decentralization from national to sub-national levels can also improve responsiveness to local needs and priorities, further strengthening health systems (WHO, 1978, 2007; Kuruvilla *et al*., 2014; Mkoka *et al*., 2014; Maluka and Bukagile, 2016; Tsofa *et al*., 2017). Moreover, giving the community a voice in HGL promotes ownership, utilization of services and better health outcomes (WHO, 1978, 2008; Cornwall *et al*., 2000; Tsofa *et al.*, 2017). In contrast, over-centralization of HGL, vertical programming and misalignment between partners, national priorities and local needs resulted in inefficient service delivery in Kenya and Zimbabwe.

Accountability is a critical responsibility of national health leaders who must establish and implement mechanisms to monitor, review and act on results to improve child survival (WHO, 2011, 2015; Mishra *et al*., 2015; Schweitzer, 2015). In line with the global *Countdown to 2015* expectations that countries monitor coverage of recommended MNCH interventions, identify gaps and propose new actions to improve survival (Bellagio Group, 2003; Bryce *et al*., 2006; Victora *et al*., 2016), both Liberia and Zambia were sharply focused on progress towards MDG#4 and quickly responded to deficiencies by implementing appropriate policies, strategies and initiatives. These strategic reforms were facilitated through a well-functioning HMIS (WHO, 2007) and a robust data-driven M&E approach, as has been shown in other LMICs that have met MDG#4 (Rowe, 2009; Kuruvilla *et al*., 2014). Although *Countdown* has raised the visibility and accountability for MNCH worldwide, many LMICs including Kenya and Zimbabwe lack sufficient data on vital statistics, disease surveillance, resource utilization or service availability to inform appropriate responses (Grove *et al*., 2015; Mikkelsen *et al*., 2015). Further investments are needed to ensure that MNCH data are collected at the point of care, transferred between health system levels, and compiled and reported at both national and local levels (AbouZahr *et al*., 2010; Agyepong *et al*., 2018). 

A major strength of this study is the comparison of two SSA nations that achieved MDG#4 with two that did not, highlighting successful strategies and persistent challenges influencing U5M. We conducted an extensive document review and obtained qualitative data from diverse participants. Limitations of our methods for the individual case studies have been published (Kipp *et al*., 2016; Brault *et al*., 2017, 2018; Haley *et al*., 2017). Because we were evaluating progress towards MDG#4 which measures U5M, we focused on pregnancy, the newborn period and early childhood, though we recognize that the continuum now also includes reproductive and adolescent periods (Countdown to 2030 Collaboration, 2018). Use of only four countries limits the study’s generalizability across SSA; however, our findings corroborate and extend findings from other countries that have successfully reduced U5M.

Strong HGL can drive a significant reduction in U5M despite considerable financial, social and political challenges (Kuruvilla *et al*., 2014; Mishra *et al*., 2015). Political and health leaders must prioritize child survival on their development agendas, engage and align partners with national activities and commit adequate resources for universal availability of MNCH services (Bryce *et al*., 2013; Every Woman Every Child, 2015; United Nations, 2015). Cross-sector policies and strategies should concurrently address all determinants of MNCH, tackle inequities in access and quality of care, and encourage accountability (Agyepong *et al*., 2018). The experiences from our study countries can contribute to attaining the Sustainable Development Goal target of reducing U5M rates to <25 per 1000 live births in each country by 2030 (United Nations, 2015).

## Ethics

The Institutional Review Board at Vanderbilt University Medical Center approved the qualitative component of the study, with Vanderbilt serving as the Coordinating Center (IRB# 130567). Local ethics approval was obtained from the following committees prior to data collection: Kenyatta National Hospital Ethics & Research Committee (KNH-ERC/A/A259; Kenya), University of Liberia Office of the Institutional Review Board (Liberia), ERES Converge Institutional Review Board (IRB# 00005948; Zambia), Joint Parirenyatwa Hospital and University of Zimbabwe College of Health Sciences Research Ethics Committee (JREC/193/13; Zimbabwe) and the Medical Research Council of Zimbabwe (MRCZ/A/1772; Zimbabwe).

## Supplementary Material

Supplementary Data
