# Supplementary material for: Promoting progress in child survival across four African countries: the role of strong health governance and leadership in maternal, neonatal and child health
Source: Health Policy Plan. 2019 Jan 29;34(1):24–36. doi: 10.1093/heapol/czy105 (PMC6479825; doi:10.1093/heapol/czy105)
Supplement: Supplementary Data [file czy105_supp.zip › czy105-Suppl_data/czy105_Suppl_Table_2.pdf]

Table 2. Selected study sites within Kenya, Liberia, Zambia, and Zimbabwe.

**Country**	**Capital**	**Urban**	**Rural**
Kenya*	Nairobi *(Nairobi Province)*		Embu *(Eastern Province)*
Liberia	Monrovia *(Montserrado County)*		Gbarnga *(Bong county)*
Zambia	Lusaka	Livingstone *(Southern Province)*	Kazungula *(Southern Province)*
Zimbabwe	Harare	Chinhoyi *(Mashonaland West Province)*	Banket *(Mashonaland West Province)*

*Nairobi Province is now Nairobi County; Eastern Province now consists of 8 counties (established in 2013), including Embu County as the rural study site.
