# Supplementary material for: Promoting progress in child survival across four African countries: the role of strong health governance and leadership in maternal, neonatal and child health
Source: Health Policy Plan. 2019 Jan 29;34(1):24–36. doi: 10.1093/heapol/czy105 (PMC6479825; doi:10.1093/heapol/czy105)
Supplement: Supplementary Data [file czy105_supp.zip › czy105-Suppl_data/czy105_Suppl_Table_4.pdf]

Table 4: Numbers of key informants interviewed for each country

	Ministry of Health	Donor Organization	Community Based Organization	Health Care Worker	Total
Kenya	9	8	13	13	43
Liberia	11	8	14	14	47
Zambia	6	6	10	9	31
Zimbabwe	6	6	6	12	30
Total	32	28	43	48	151
