# Supplementary material for: Promoting progress in child survival across four African countries: the role of strong health governance and leadership in maternal, neonatal and child health
Source: Health Policy Plan. 2019 Jan 29;34(1):24–36. doi: 10.1093/heapol/czy105 (PMC6479825; doi:10.1093/heapol/czy105)
Supplement: Supplementary Data [file czy105_supp.zip › czy105-Suppl_data/czy105_Suppl_Table_5.pdf]

Table 5. Comparison of Health Governance and Leadership elements between progressing and non-progressing countries.

	Progressing		Not progressing	
	Liberia	Zambia	Kenya	Zimbabwe
**Prioritization and Support of Child Survival**				
Political support	+	+	+/-	+/-
Current policy framework	+	+	+	-
Policies and strategies implemented	+	+	-	-
Concurrent national policy focus on health, social welfare, development	+	+	-	-
Triple planning approach	+	+	+/-	-
Abuja Declaration target met during study	+	+	-	-
Non-financial health system resources (e.g. human, material, facility, etc.)	+	+	-	-
**Collaboration, Coordination and Inclusion**				
Donors aligned with national priorities	+	+	-	-
Collaborative strategic planning with partners/stakeholders	+	+	-	-
Coordination/collaboration between health and other sectors	+	+	-	-
Coordination and sharing resources among different health programs	+	+	-	-
Coordination of MNCH services across health system levels	+	+	-	-
Integrate packages of health services at point of care	+	+	-	-
Decentralization of decision-making and resource allocation	+	+	-	-
Beneficiaries included in strategic planning (community input)	+	+	-	-
**Accountability**				
Clear roles, responsibilities and expectations	+	+	+/-	-
Updated, effective HMIS	+	+	-	-
Consistent data collection and reporting at all health system levels	+	+	-	-
Ongoing monitoring and evaluation of health programs and interventions	+	+	-	-
Specifically monitoring of progress towards MDG#4	+	+	+/-*	+/-
Data-driven planning and decision making responsive to population needs	+	+	-	-
Local involvement (Community planning boards and committees)	+	+	-	-

\+ Indicates clear activity, policy, participation, and/or implementation of an element in the defined area during the study period

\- Indicates a lack of engagement of this element or merely planning, but not implementing policy/action during the study period
N/A Indicates not available

+/- Indicates ambiguous activity, policy, participation, and/or implementation of an element in the defined area

*Note: We found information indicating that a Kenya Country Countdown was conducted in 2013 (end of the study period), though this was not reported to our study team by Kenya’s MOH.
