# Supplementary material for: Promoting progress in child survival across four African countries: the role of strong health governance and leadership in maternal, neonatal and child health
Source: Health Policy Plan. 2019 Jan 29;34(1):24–36. doi: 10.1093/heapol/czy105 (PMC6479825; doi:10.1093/heapol/czy105)
Supplement: Supplementary Data [file czy105_supp.zip › czy105-Suppl_data/czy105_Suppl_Figure_1.pdf]

Figure 1. Trends in under-five mortality and progress towards Millennium Development Goal #4 for Liberia, Zambia, Kenya, and Zimbabwe, 1990-2015.

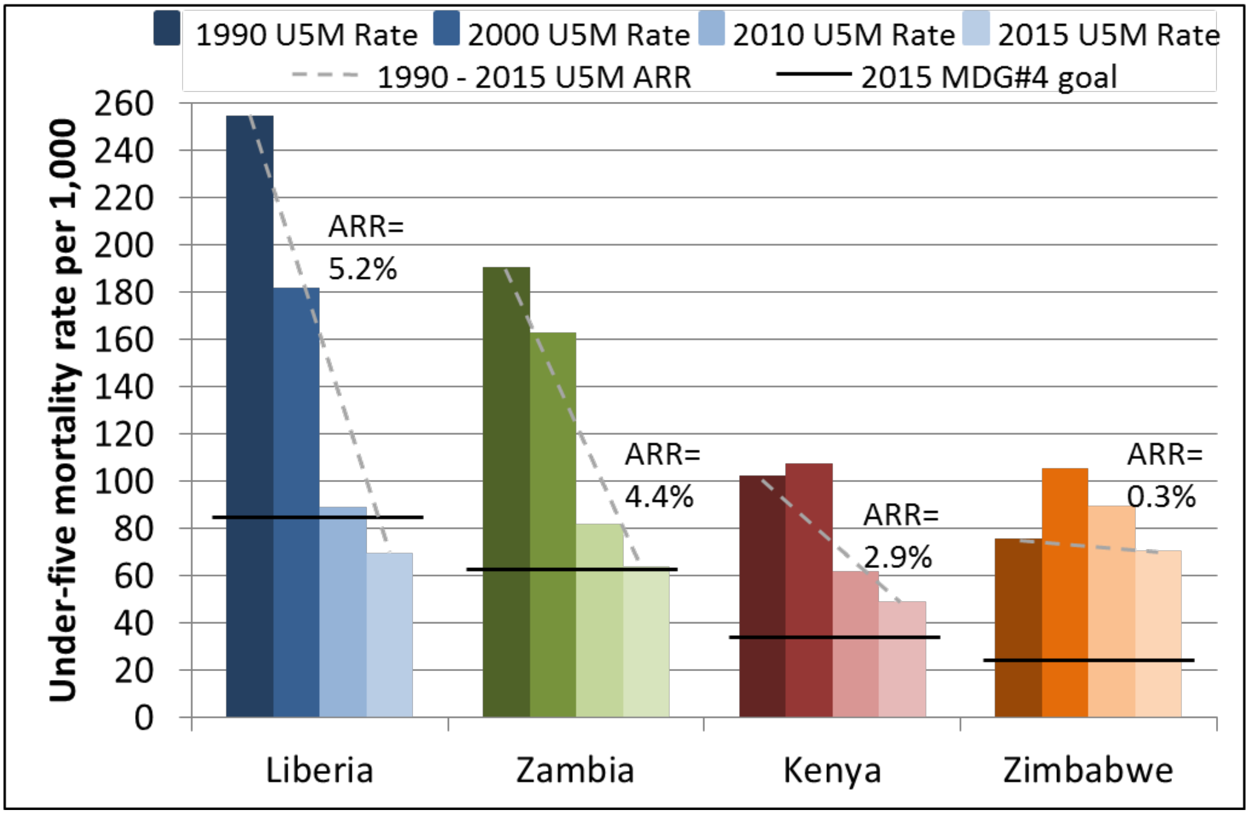

Source: *Levels and Trends in Child Mortality: Report 2015 - Estimates Developed by the United Nations Inter-agency Group for Cild Mortality Estimation (UNICEF et al., 2015)*

U5M, under-five mortality, the deaths among children under age 5 years per 1000 live births; ARR, annual rate of reduction; MDG, Millennium Development Goal.
