# Supplementary material for: Promoting progress in child survival across four African countries: the role of strong health governance and leadership in maternal, neonatal and child health
Source: Health Policy Plan. 2019 Jan 29;34(1):24–36. doi: 10.1093/heapol/czy105 (PMC6479825; doi:10.1093/heapol/czy105)
Supplement: Supplementary Data [file czy105_supp.zip › czy105-Suppl_data/czy105_Suppl_Boxes.pdf]

Box 1: Historical and contextual factors impacting Liberia during the study period

- Located on the western coast of Africa with a small population of about 3.5 million, half of whom reside in urban areas
- Fourteen years of civil war ending in 2003 destroyed most of the national infrastructure, internally displaced many people, and cost at least 200,000 lives
- First democratic election held in 2005 began a new phase of national reforms and rebuilding
- Prudent macroeconomic management, social stability, and substantial foreign investments have facilitated efforts to overcome the civil crisis and re-establish its economy
- Economic growth affected by infrastructure constraints, unemployment, a narrow base of the economy, and the country's dependency on food and fuel imports
- Compounding challenges include flooding and drought in some areas, outbreaks of communicable diseases, influx of more than 150,000 refugees from neighboring Cote D'Ivoire, and increasing dependence on international aid

Box 2: Historical and contextual factors impacting Zambia during the study period

- Gained independence in 1964, has since enjoyed decades of political stability and freedom from conflict enabling a consistent focus on development and reforms
- Experienced consistent economic growth and strong macroeconomic indicators over several decades
- Growing population, high level of urbanization, and increasing life expectancy
- Young and increasing population also intensifies the burden of health needs on the economy
- Economic growth has not translated into significant poverty reduction at household level; More than half of the population lives below the poverty line, most considered to be in extreme poverty
- Unemployment has been high and income inequity is significant

Box 3: Historical and contextual factors impacting Kenya during the study period

- Largest and most diversified economy in East Africa
- Strategically located to serve as an important transport hub for much of Eastern Africa
- Large and growing population consisting of most major ethno-racial and linguistic groups found in Africa
- High absolute poverty; more than two-thirds of urban population living in slums
- Violence following the 2007 presidential elections worsened mistrust between different political and ethnic groups
- Decades of globalization, political instability, regional and national macroeconomic challenges, and climate change have contributed to high inequities

Box 4: Historical and contextual factors impacting Zimbabwe during the study period

- Overcame a decade of civil war to gain independence in 1980, and successfully established one of the strongest economies and health systems in southern Africa
- Long period of relative stability and progress following independence until it experienced a drastic economic decline and hyperinflation beginning in the late-1990's

- Prior to the economic crisis, Zimbabwe had a highly performing health delivery system supporting a long track record of delivering comprehensive health services across the country.
- Nearly a quarter of the population left the country, including a large proportion of the workforce
- High poverty rates, unemployment and food insecurity persisted during the study period
- Despite challenges, education and literacy rates remained high among both men and women
