# Supplementary material for: Promoting progress in child survival across four African countries: the role of strong health governance and leadership in maternal, neonatal and child health
Source: Health Policy Plan. 2019 Jan 29;34(1):24–36. doi: 10.1093/heapol/czy105 (PMC6479825; doi:10.1093/heapol/czy105)
Supplement: Supplementary Data [file czy105_supp.zip › czy105-Suppl_data/czy105_Suppl_Table 3.pdf]

Table 3: Additional inclusion criteria for each key informant group

Key Informant Type	Description
All participants	- Age 18 years or older<br>- Have adequate knowledge or experiences related to childhood survival specified for each participant group below<br>- Speak English or the most common local language,<br>- Able to provide written or verbal informed consent.
Ministry of Health	- National or provincial-level officials working in government-level health care system administration, policy-making, program development, or leadership.<br>- All officials working in areas related to MNCH were eligible.
Donor Partners	- Individuals working as directors, managers, or other leaders of entities providing financial or other aid for MNCH services, or serving as the implementing partner.<br>- International or national organizations focusing entirely on MNCH or with MNCH as one component of their mission.<br>- Organizations had to be officially registered in the country.
Members of Community-Based Organizations	- Directors, leaders, managers working for a CBO involved in or providing referrals to MNCH services within the study site.<br>- Organizations had to be officially registered in the country.
Health Care Providers	- Professionally trained physicians, nurses, clinical officers, or other health-related staff such as environmental health technicians, pharmacists, or community health workers.<br>- Working in a health facility providing MNCH care.
