# Supplementary material for: Promoting progress in child survival across four African countries: the role of strong health governance and leadership in maternal, neonatal and child health
Source: Health Policy Plan. 2019 Jan 29;34(1):24–36. doi: 10.1093/heapol/czy105 (PMC6479825; doi:10.1093/heapol/czy105)
Supplement: Supplementary Data [file czy105_supp.zip › czy105-Suppl_data/czy105_Supp_Table_1.docx]

Table 1. Key questions and deductive themes explored during the review of national health policies and strategies and key informant interviews that cut across child survival content areas.

| **Specific questions for review of national policies and strategies** | **Specific themes explored across content areas with key informants** |
| --- | --- |
| What policies and strategies related to MNCH health were in place between 2000 and 2013 (including changes during this period)?  What challenges were stated as hindering progress towards MDG#4?  What facilitators were stated as enabling progress towards MDG#4?  What changes or improvements to MNCH policies and strategies were proposed or newly implemented towards the end of the study period but were not yet measurable? | Issues related to program evaluation, access and utilization, coverage, impact, and sustainability, as appropriate  Knowledge and experiences related to MNCH across the *health care continuum* (prenatal care through age 5 years)  Knowledge and experiences related to MNCH across the *health system continuum* (community to tertiary hospitals) |
